# Glutamine ameliorates hyperoxia-induced hippocampal damage by attenuating inflammation and apoptosis *via* the MKP-1/MAPK signaling pathway in neonatal rats

**DOI:** 10.3389/fphar.2023.1096309

**Published:** 2023-02-02

**Authors:** Chouhui Xuan, Haixia Cui, Zhengyong Jin, Yuyang Yue, Shuxia Cao, Songbiao Cui, Dongyuan Xu

**Affiliations:** ^1^ Department of Pediatrics, Yanbian University Hospital, Yanji, Jilin, China; ^2^ Department of Clinical Laboratory, Yanbian University Hospital, Yanji, Jilin, China; ^3^ Department of Dermatology, Yanbian University Hospital, Yanji, Jilin, China; ^4^ Department of Center of Morphological Experiment, Yanbian University, Yanji, Jilin, China; ^5^ Department of Neurology, Yanbian University Hospital, Yanji, Jilin, China; ^6^ Key Laboratory of Cellular Function and Pharmacology of Jilin Province, Yanbian University, Yanji, China

**Keywords:** glutamine, hyperoxia, hippocampus, learning and memory impairment, inflammation, MKP-1, MAPK

## Abstract

Glutamine (Gln) is an immunomodulatory protein that mediates oxidative stress, inflammation, and apoptosis, but has not been reported in the treatment of hyperoxia (Hyp)-induced brain injury. The aim of this study was to determine whether Gln could improve hyp-induced brain injury in neonatal rats to and later learning and memory dysfunction, and to explore its possible mechanisms. We prepared a model of neonatal rat brain injury caused by normobaric hyperoxia while administered with Gln for 7 days for evaluation. Learning memory function was assessed with the Morris water maze test. Histological analysis, protein expression analysis, oxidative stress and inflammation level analysis were performed using hippocampal tissue. Gln treatment significantly reduced brain tissue water content, oxidative stress levels, microglia activation and inflammatory factor expression, and attenuated tissue damage and apoptosis in the hippocampal region. Gln ameliorates hyp-induced learning, memory impairment in neonatal rats in water maze test. It also increased MKP-1 protein expression and decreased p-p38, p-ERK and p-JNK. Therefore, it is hypothesized that Gln may exert neuroprotective effects by increasing MKP-1 expression to negatively regulate MAPK signaling, with potential cognitive improvement in hyp-induced brain injury.

## Introduction

Gln, an L-alpha-amino acid, is the most abundant non-essential amino acid in the body. It is an important nutrient for the conversion of some amino acids and biomolecules and is frequently used as a source of energy for some rapidly dividing cells (Hu et al., 2020). Gln is used clinically as an immunotropic modulator (Kim, 2011) in the treatment of various diseases such as hyp-induced acute lung injury (Perng et al., 2010), traumatic brain injury, bronchial asthma, and ulcerative colitis. Gln stores are depleted and rapidly released in the presence of stressors, causing a decrease in intracellular Gln levels. Supplementation of Gln *via* an external source increases expression of glutathione, which in turn reacts directly with reactive oxygen species (ROS) to prevent oxidative damage (Oliveira et al., 2010), effectively reduces ROS and inflammatory factor levels (Gong et al., 2017), and promotes neurotrophic factor expression and neurogenesis, thereby improving cell survival.

Oxygen therapy is one of the most common and important treatments used in the resuscitation of preterm infants. However, at the critical stage of life, increased oxygen levels may affect the process of brain development. ROS are formed in excess during oxygen therapy, causing oxidative stress and leading to cellular inflammation and apoptosis (Sifringer et al., 2013); neuronal development is also adversely affected (Al et al., 2020). Preterm infants are particularly susceptible to ROS-induced damage as their antioxidative systems are not completely developed at birth, and during childhood, these infants are at a tenfold risk of developing cognitive and learning disabilities as well as behavioral abnormalities (Hintz et al., 2018). Studies have shown that early postnatal intervention *via* administration of Gln may increase the volume of cerebral structures, improve brain development in preterm infants, and reduce occurrence of adverse outcomes such as neurocognitive dysfunction and attention deficit hyperactivity disorder (ADHD) in later life (de Kieviet et al., 2014). Therefore, it is necessary to develop a safe and effective neuroprotective drug for preterm infants with hyp-induced brain injuries.

MAPK pathway is increasingly recognized as playing a key role in regulating ROS production (Lv et al., 2020). Phosphatase-1, or MKP-1 (dual specificity phosphatase 1, also known as DUSP1), inactivates this pathway and is essential for the control of inflammation. MKP-1 not only suppresses inflammation by dephosphorylating the MAPK family of proteins at key modulation sites, but is also involved in negative feedback regulation and homeostatic function in cellular transduction. Regulation of the activity of related factors in the MKP-1/MAPK signaling pathway may be critical for preventing and counteracting neuroinflammation and apoptosis. Studies have shown that the anti-inflammatory and anti-apoptotic effects of Gln in acute lung injury are mediated by the MAPK signaling pathway (Huang et al., 2021). Additionally, Gln regulates the MAPK pathway *via* upregulation of MKP-1, which balances the inflammatory response (Kim et al., 2022).

To test the hypothesis that Gln may regulate the MKP-1/MAPK pathway and cause a decrease in oxidative stress, inflammation, and apoptosis, thereby preventing brain damage, learning difficulties and memory dysfunction caused by hyp-induced injury, in this study we investigated the protective role of Gln in hyp-induced brain injury in neonatal rats and its association with the MKP-1/MAPK signaling pathway using a neonatal rat brain subjected to normobaric hyperoxia as a model.

## Material and methods

### Main reagents

MKP-1 (#sc-373841), p-p38 (#sc-166182), p38 (#sc-81621), p-JNK (#sc-6254), JNK (#sc-7345), p-ERK (#sc-7383), ERK (#sc-135900), BDNF (#sc-65514), Synapsin Ia (#sc-376623), MBP (#sc-376995), Bax (#sc-20067), Bcl-2 (#sc-7382) antibodies were purchased from Santa cruz biotechnology (United States). Goat anti-rabbit IgG/horseradish enzyme labeling (#16K22C) was purchased from Boster Biological Technology (Wuhan, China), and goat anti-mouse IgG/horseradish enzyme labeling (#ZB-2305) was purchased from Zhongshanjinqiao biotechnology (Beijing, China). β-actin antibody (bsm-33036m) (1:1000) was purchased from Beijing Boaosen Biotechnology Co., Ltd. (Beijing, China). L-glutamine (G8540) was purchased from Sigma-Aldrich (United States). Rat IL-1β (SBJ-H0417), IL-6 (SBJ-H0465), TNF-α (SBJ-H0038) ELISA kit reagent kits were purchased from Nanjing Sempega Biotechnology Co. (Nanjing, China). ROS (MM-43700M2), GSH-PX (MM-20251R2), SOD (MM-20387R2), MDA (MM-0385R1) ELISA kits were purchased from Jiangsu Meimian Industrial Co., Ltd. (Yancheng, China). TUNEL kit was purchased from Promega (United States). H-E staining kit was purchased from Beijing Solarbio Science and Technology Co., Ltd. (Beijing, China). Iba-1 antibody was purchased from Santa cruz biotechnology (United States). DAB kit was purchased from Beijing Boaosen Biotechnology Co., Ltd. (Beijing, China).

### Animals and experimental procedures

SD neonatal rats, weighing 6.7–8.2 g, were used in this study, provided by the Animal Section of Yanbian University. All animals were kept together with their dams in per cage on a 12 h light/dark cycle in a temperature-controlled room (22°C ± 1°C) with free access to water and food.

60 newborn rats were randomly divided into four groups (*n* = 15) 1 day after birth: control group (Con: 21% O_2_), normal drug group (Con + Gln: 21% O_2_), Hyp group (Hyp: 85% O_2_) and drug group (Hyp + Gln: 85% O_2_). The Hyp and Hyp + Gln groups were placed in glass chambers and oxygen concentrations were measured twice daily with a digital oximeter, and nitrogen dioxide and water were absorbed with soda lime. The dams were rotated between the hyperoxia- and normoxia-exposed pups every day to avoid undersupply of the young. Gln was dissolved in 0.9% Nacl to be used. Gln (1 μg/g B.W) was administered intraperitoneally to newborn rats in the Con + Gln and Hyp + Gln groups at the same time each day, and the same volume of 0.9% NaCl was administered intraperitoneally to newborn rats in the Con and Hyp groups at the same time for 7 days.

**FIGURE 1:**
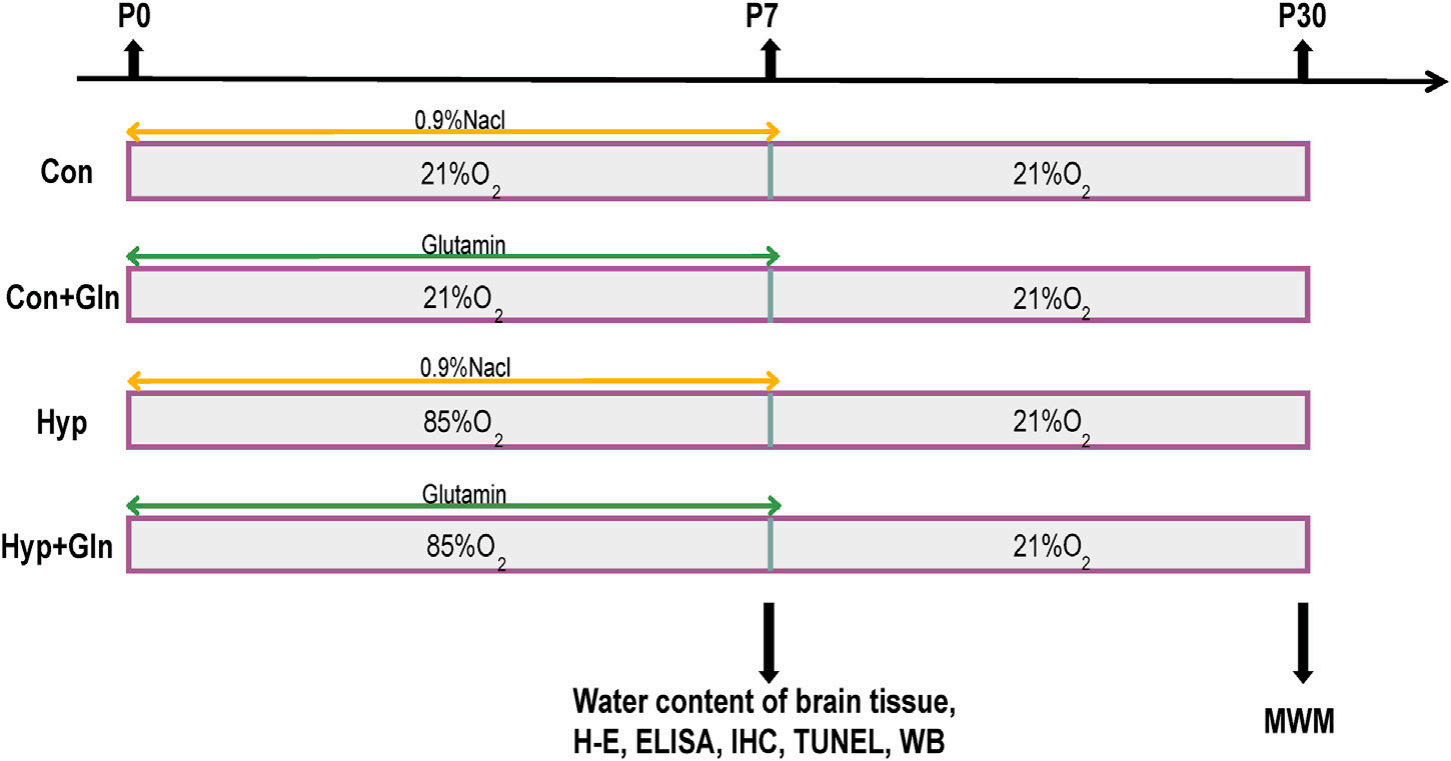
The experimental procedure. Newborn rats were randomly divided into four groups: Con, Con + Gln, Hyp and Hyp + Gln. The Hyp and Hyp + Gln groups were placed in 85% O_2_ for 7 days, Con and Con + Gln groups were placed indoor (21% O_2_). Gln [1 μg/g (B)W] was administered intraperitoneally to newborn rats in the Con + Gln and Hyp + Gln groups at the same time each day, and the same volume of 0.9% NaCl was administered intraperitoneally to newborn rats in the Con and Hyp groups at the same time for 7 days. At P7, each group of randomly selected newborn rats for histopathological analysis (*n* = 3), determine the water content of brain tissue (*n* = 3) and protein analysis (*n* = 3). At P30, each group of neonatal rats (*n* = 6) began the Morris water maze experiment. Con, control; Hyp, hyperoxia; Gln, glutamine; P0, postnatal day 0; P7, postnatal day 7; P30, postnatal day 30; IHC, immunohistochemistry; WB, western blot; MWM, morris water maze experiment.

At P7, each group of randomly selected newborn rats (*n* = 3) was anesthetized, fixed on the operating table, and the thoracic cavity was exposed. After injection of chilled PBS solution from the apex of heart, the tissue was fixed by injection of 4% paraformaldehyde. The brain tissue was then quickly removed, immersed in 4% paraformaldehyde solution for 24 h and fixed, and subjected to histopathological analysis (HE staining, immunohistochemistry, TUNEL staining) after tissue pruning, dehydration, transparency, paraffin embedding and conventional sectioning. In each group, newborn rats (*n* = 3) were randomly selected and executed after anesthesia, and a pair of hippocampal tissues were rapidly isolated on ice, treated with liquid nitrogen and placed in an ultra-low temperature refrigerator at −80°C for backup (ELISA, Western Blot). And each group of newborn rats (*n* = 3) was then randomly selected and executed after anesthesia, and brain tissue was removed to determine the water content of brain tissue. At P30, each group of neonatal rats (*n* = 6) began the Morris water maze experiment (Figure 1).

### Brain water content measurement

Newborn rats were selected at random from each group (*n* = 3), and following ether anesthesia, the brain tissues were immediately removed and weighed. The wet weight (W) of the brain tissues was marked and baked in a 60°C oven. The dry brain tissue weight (D) is measured when there is no weight change for two consecutive days. The brain tissue water content (%) was determined using the formula [(W-D)/W] 100%.

### Assay for oxidative stress parameters

Hippocampal tissues were removed and washed with pre-cooled saline, dried on filter paper. The hippocampal samples with saline solution were homogenized at a ratio of 1:9 (g/mL) in a homogenizer, and the supernatant was collected by centrifugation at 3,000 rpm for 10 min. *Hippocampus* oxidation index was determined using malondialdehyde (MDA), ROS, superoxide dismutase (SOD) and glutathione (GSH) Elisa kits. The MDA level was measured by the absorbance of the sample at 532 nm, expressed as nmol/mg protein. The SOD level was measured by the absorbance of the sample at 550 nm, expressed as unit/mg protein. The ROS level was measured by the absorbance of the sample at 525 nm, expressed as RFU/mg protein. The GSH level expressed as ng/mg protein. All results were normalized by the total protein concentration in each sample.

### ELISA assay

The hippocampal samples with saline solution were homogenized at a ratio of 1:9 (g/mL) in a homogenizer, and the supernatant was collected by centrifugation at 3,000 rpm for 10 min. The expression levels of TNF-α, IL-1β and IL-6 in hippocampus tissue were determined using quantitative ELISA kits according to the manufacturer’s instructions.

### Morris water maze (MWM) test

To observe the ability to reflect spatial learning and memory, the MWM test was conducted at P30-P34. The Morris water maze apparatus consisted mainly of a 160 cm-wide, 60 cm-high circular pool with a black interior and a water level 30 cm deep, which was kept at 20°C ± 1°C. A cylindrical platform with a diameter of 12 cm wide was set 1 cm below the water surface of the third quadrant. In the first phase, spatial learning ability was tested (localization-navigation test): each rat was tested four times a day (with a 2-min break in between) for 4 days. In each test, newborn rats (*n* = 6) were placed in the water facing the wall of the tube from quadrants 1 to 4 sequentially, swam for a maximum of 2 min to find the platform, and remained on the platform for 20 s. If the platform was not found within 2 min, they were guided to the platform for 20 s and the escape latency was recorded as 2 min. In the second phase, spatial memory was examined (spatial exploration test): the platform was removed and the rats were all placed in the water along the pool wall from the same quadrant and the swimming time and distance in the target quadrant and the number of times they crossed the platform in 2 min were recorded. The experimental process was recorded and analyzed by the data acquisition system.

### Histopathological assessment of brain injury

The brain tissues were fixed in 4% PFA solution for 24 h, and then dehydrated with graded alcohol. Brain tissue was embedded in paraffin and sliced at a thickness of 5–7 μm. H&E staining kit was used according to the instructions. Finally, the sections were sealed with neutral gum.

### TUNEL staining

Brain tissue sections were dewaxed and treated with proteinase K working solution to permeabilize the cell and nuclear membranes and 3% H_2_O_2_ methanolic closure solution. The sections were incubated with TUNEL reaction mixture for 1 h at 37°C protected from light, and then reacted with anti-fluorescein antibody for 30 min protected from light. The sections were then incubated for 5 min using a 4, 6-diamidino-2-phenylindole (DAPI) (Santa Cruz, United States) solution. The apoptotic cells were quantitatively assessed, with three animals examined per group and three slices per hippocampal sample. TUNEL-positive hippocampal neurons of the CA1 zone were identified and counted under ×200 magnification, and the average number of positive apoptotic hippocampal neurons in the CA1 of three brains in a group was calculated.

### Immunohistochemical staining

The slides underwent dewaxing, dehydration, rehydration, antigen repair in boiling sodium citrate buffer, cold confirmed to room temperature and then rinsed in PBS. After 15 min of endogenous peroxide blocker blockade, the slides incubated with primary antibody Iba-1 (1:500) overnight at 4°C. After rewarming at room temperature for 1 h, PBS rinsing, they were then incubated with secondary antibodies (anti-goat anti-rabbit; Servicebio, Wuhan, China) at room temperature for 30 min. PBS rinsing them again and DAB color development for 5 min. The CA1 and CA3 regions of the hippocampus were analyzed. The images (200x, 400x) were captured by the microscope system (Leica, Germany). The experiment was repeated three times. The average number of activated microglia in the CA1 and CA3 area of three hippocampal in a group was calculated under ×400 magnification.

### Western blotting

To investigate the expression of proteins by western blot analyses, hippocampal tissue samples were lysed in a protein cell lysis buffer (RIPA buffer (Solarbio, #R0010, Beijing, China): PMSF (Solarbio, #P0100, Beijing, China): phosphatase inhibitor (BestBio, #BB 1907, Shanghai, China) = 100:1:1). The protein concentration of the samples was determined using a bicinchoninic acid (BCA) protein assay kit (Thermo Scientific, #1859078, Shanghai, China). The samples were boiled for 10 min, and proteins were separated by electrophoresis using a 10% or 12% sodium dodecyl sulfate (SDS)-polyacrylamide gel. Then, proteins transferred to PVDF membranes (SigmaAldrich, #PR05505, United States). The membrane was blocked with 5% skim milk (diluted with TBST), incubated with primary antibody overnight at 4°C. The membrane was washed three times with 0.1% TBST, and incubated with secondary antibody for 1.5 h and then washed again with TBST. Then, the membranes were detected with an enhanced chemiluminescence kit (Boster, 16K22C02, China). The samples were wrapped in plastic wrap and transferred into the dark room. The processes of developing and fixing were done after the exposure of X-ray film. The results were scanned by a gel imaging system and analyzed by using ImageJ software. The grayscale value of each band was normalized to the grayscale value of the β-actin band, and the relative expression of the proteins was determined. The primary antibody included MKP-1 (1:1000), p-p38 (1:1000), p38 (1:1000), p-JNK (1:1000), JNK (1:1000), p-ERK (1:1000), ERK (1:1000), BDNF (1:1000), Synapsin Ia (1:1000), MBP (1:1000), Bax (1:1000), Bcl-2 (1:1000). The secondary antibodies include Goat anti-rabbit IgG/horseradish enzyme labeling (1:5000) and goat anti-mouse IgG/horseradish enzyme labeling (1:5000).

### Statistical analysis

SPSS26.0 software was used for statistical analysis, and GraphPad Prism 8.0.1 software was used to crate figures. Values are presented as mean ± standard deviation, and statistical significance was determined by one-way ANOVA followed by Tukey and Bonferroni tests. Univariate repeated measures ANOVA was used to analyze escape latency (MWM) for independent group comparisons. *p* < 0.05 was considered statistically significant.

## Result

### Gln ameliorates hyp-induced elevated brain water content and hippocampal histopathological changes

**FIGURE 2:**
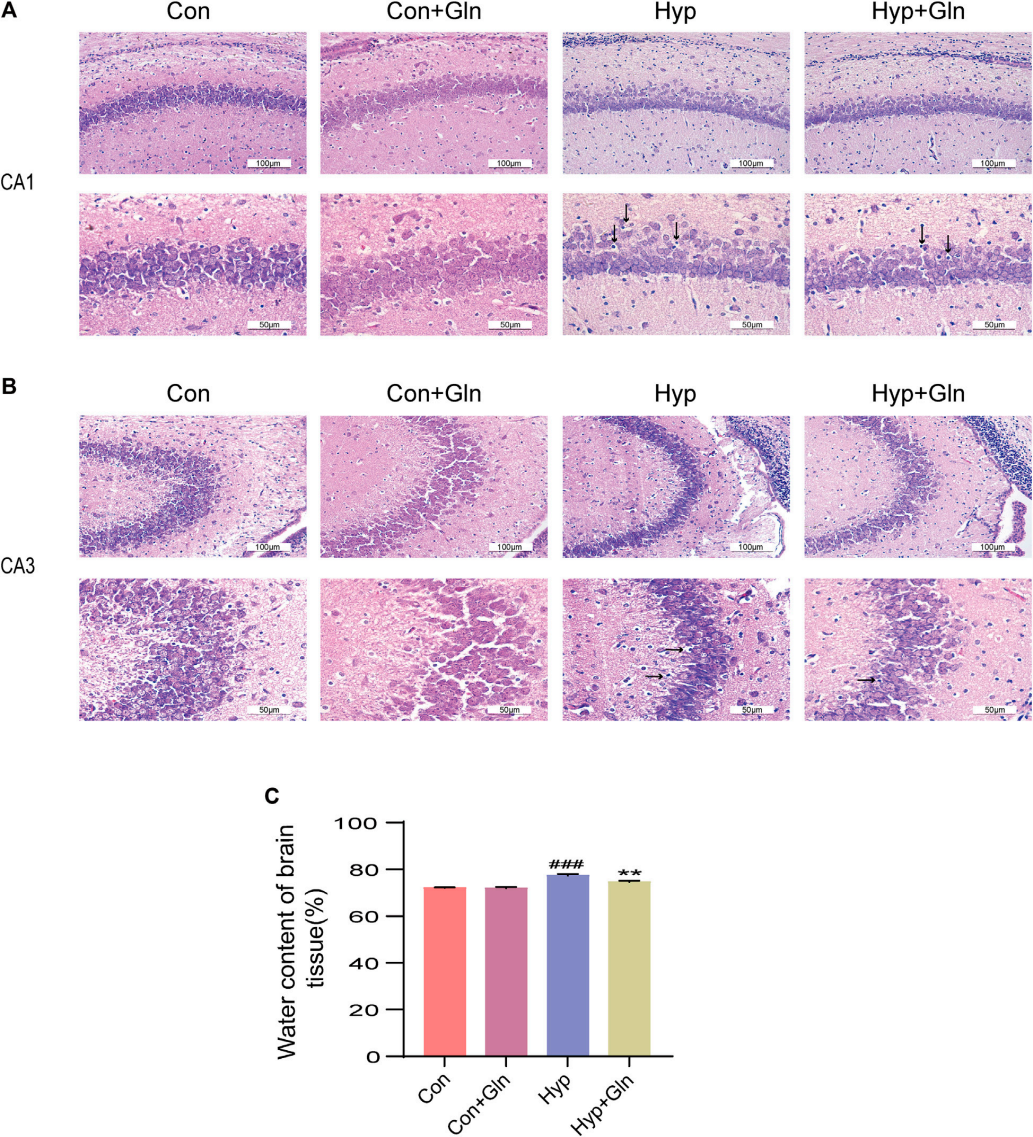
Gln ameliorates hyperoxia-induced cerebral edema and brain tissue damage. **(A)** Morphological changes of CA1 area of hippocampus in each group. Scale bar = 100 µm, 50 µm **(B)** Morphological changes of CA3 area of hippocampus in each group. Scale bar = 100 µm, 50 µm **(C)** Changes of brain water content in each group. Con (72.11 ± 0.12), Con + Gln (71.94 ± 0.32), Hyp (77.41 ± 0.37), Hyp + Gln (74.66 ± 0.29). ^###^ *p* < 0.001 vs. the Con, **p < 0.01 vs. the Hyp (*n* = 3). *p* < 0.05 was considered statistically significant. Areas of severe histopathological changes were marked by black arrows.

In this study, brain edema was observed in each group by measuring brain tissue water content. As shown in Figure 2C, the brain tissue water content in the Hyp group was significantly higher (*p* < 0.001) than that in the Con group and that in the Hyp + Gln group was significantly lower (*p* < 0.01) than that in the Hyp group. Con (72.11 ± 0.12), Con + Gln (71.94 ± 0.32), Hyp (77.41 ± 0.37), Hyp + Gln (74.66 ± 0.29) (*n* = 3).

In addition, H&E staining was used to evaluate the success of the model preparation and to determine whether Gln ameliorates hyp-induced brain tissue damage. As shown in Figures 2A, B, the cells in the hippocampal CA1 and CA3 areas of neonatal rats in the Con and Con + Gln groups had regular morphology, neat arrangement, large and round nuclei, uniform nuclei color, and clear nucleoli. Compared with the Con group, the morphology of hippocampal CA1 and CA3 pyramidal cells in the Hyp group was less regular, with solid nuclei, cytoplasmic cavities, and a more diffuse arrangement of pyramidal neurons. However, the Hyp + Gln group showed improved cell morphology and arrangement and fewer cells with nuclear fixation. These results suggest that Gln ameliorated hyp-induced brain tissue damage and edema.

### Gln improves oxidative stress after hyp-induced injury

**FIGURE 3:**
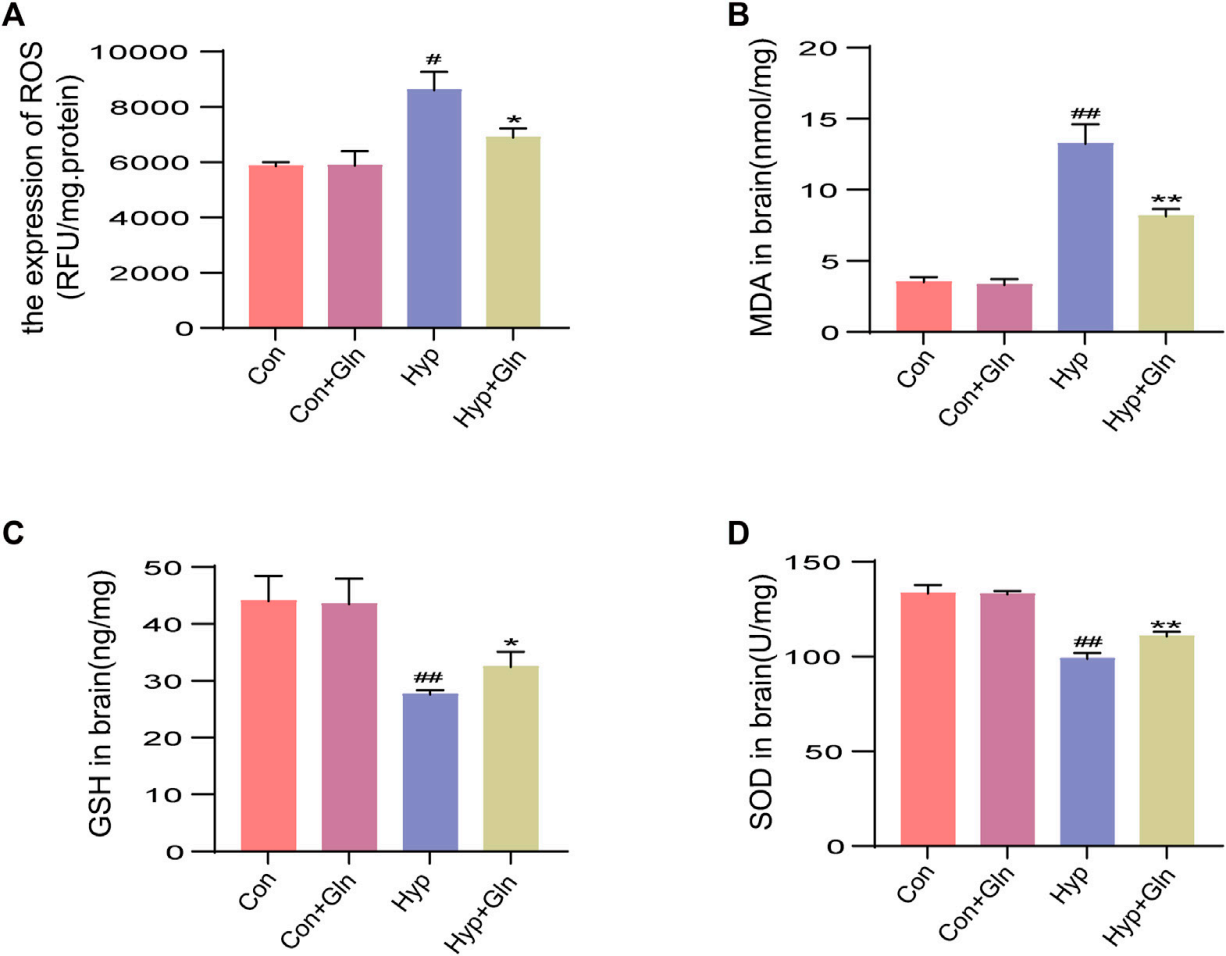
Gln improves oxidative stress after hyperoxia injury. **(A)** Total ROS content in hippocampus. Con (5851.05 ± 90.01), Con + Gln (5880.49 ± 303.02), Hyp (8160.46 ± 380.24), Hyp + Gln (6899.07 ± 184.10). **(B)** MDA content in hippocampus. Con (3.50 ± 0.21), Con + Gln (3.30 ± 0.23), Hyp (13.23 ± 0.78), Hyp + Gln (8.13 ± 0.29). **(C)** GSH content in hippocampus. Con (44.03 ± 2.54), Con + Gln (43.47 ± 2.58), Hyp (27.60 ± 0.44), Hyp + Gln (32.43 ± 1.53). **(D)** SOD content in hippocampus. Con (133.37 ± 2.49), Con + Gln (132.87 ± 1.00), Hyp (98.77 ± 1.78), Hyp + Gln (110.70 ± 1.36) (*n* = 3). ^#^ *p* < 0.05, ^##^ *p* < 0.01 vs. the Con, *p < 0.05, **p < 0.01 vs. the Hyp. *p* < 0.05 was considered statistically significant.

The supernatant of the hippocampal tissue homogenate from each group of neonatal rats was tested using relevant kits to observe changes in oxidative stress levels. The results showed that total ROS levels (*p* < 0.05) (Figure 3A) and MDA levels (*p* < 0.01) (Figure 3B) were significantly higher and GSH (*p* < 0.01) (Figure 3C) and SOD (superoxide dismutase) levels (*p* < 0.01) (Figure 3D) were considerably lower in the Hyp group than in the Con group. However, the Hyp + Gln group showed significantly lower ROS (*p* < 0.05) and MDA (*p* < 0.01) content and considerably higher GSH (*p* < 0.05) and SOD (*p* < 0.01) content than the Hyp group. These results suggest that Gln reduces the level of oxidative stress induced by hyp. ROS: Con (5851.05 ± 90.01), Con + Gln (5880.49 ± 303.02), Hyp (8160.46 ± 380.24), Hyp + Gln (6899.07 ± 184.10); MDA: Con (3.50 ± 0.21), Con + Gln (3.30 ± 0.23), Hyp (13.23 ± 0.78), Hyp + Gln (8.13 ± 0.29); GSH: Con (44.03 ± 2.54), Con + Gln (43.47 ± 2.58), Hyp (27.60 ± 0.44), Hyp + Gln (32.43 ± 1.53); SOD: Con (133.37 ± 2.49), Con + Gln (132.87 ± 1.00), Hyp (98.77 ± 1.78), Hyp + Gln (110.70 ± 1.36) (*n* = 3).

### Gln inhibits microglia activation and inflammation caused by hyp

**FIGURE 4:**
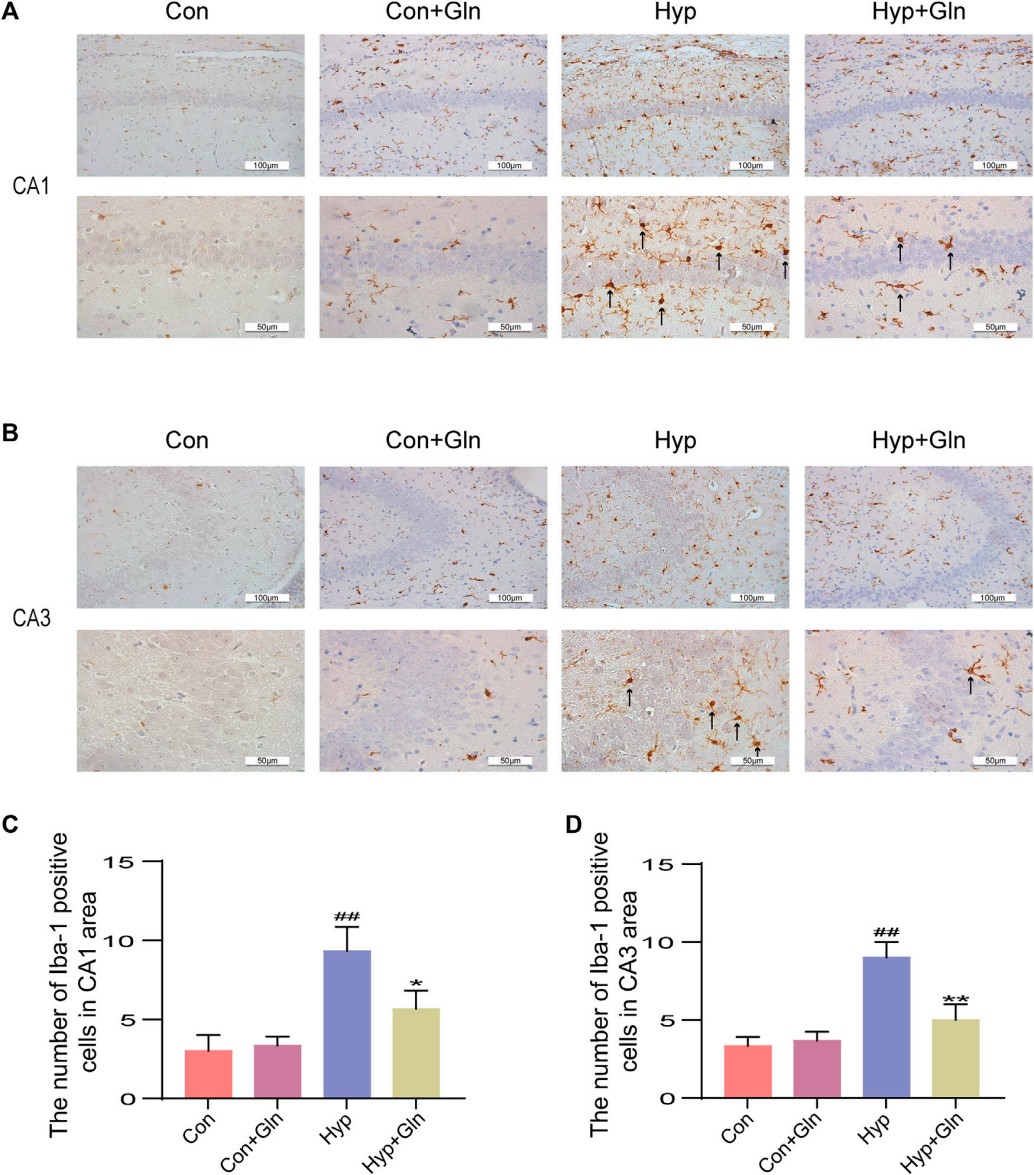
Gln inhibits microglia activation caused by hyperoxia. **(A)** Activation of microglia in the hippocampus (CA1) of each group. **(B)** Activation of microglia in the hippocampus (CA3) of each group. **(C)** Quantification of activated microglia (CA1). Con (3.00 ± 0.58), Con + Gln (3.33 ± 0.33), Hyp (9.33 ± 0.88), Hyp + Gln (5.67 ± 0.67) **(D)** Quantification of activated microglia (CA3). Con (3.33 ± 0.33), Con + Gln (3.67 ± 0.33), Hyp (9.00 ± 0.58), Hyp + Gln (5.00 ± 0.58) (*n* = 3). ^##^ *p* < 0.01 vs. the Con. *p < 0.05, **p < 0.01 vs. the Hyp. *p* < 0.05 was considered statistically significant. Activated microglia were marked by black arrows.

Microglia were marked out with Iba-1, and microglia activation in CA1 (Figures 4A, C) and CA3 (Figures 4B, D) regions of hippocampus was observed and analysed in each group. In the Con and Con + Gln groups, scattered un-activated microglia, with thin, elongated cells and few branches were observed in a resting state in the hippocampal region. In the Hyp group, microglia had enlarged cytosomes, short and thick branches and protrusions, and the number of activated cells was significantly increased (CA1: *p* < 0.01, CA3: *p* < 0.01), suggesting microglial activation. However, Gln administration significantly reduced microglial activation after hyp (CA1: *p* < 0.05, CA3: *p* < 0.01). CA1: Con (3.00 ± 0.58), Con + Gln (3.33 ± 0.33), Hyp (9.33 ± 0.88), Hyp + Gln (5.67 ± 0.67). CA3: Con (3.33 ± 0.33), Con + Gln (3.67 ± 0.33), Hyp (9.00 ± 0.58), Hyp + Gln (5.00 ± 0.58) (*n* = 3).

**FIGURE 5:**
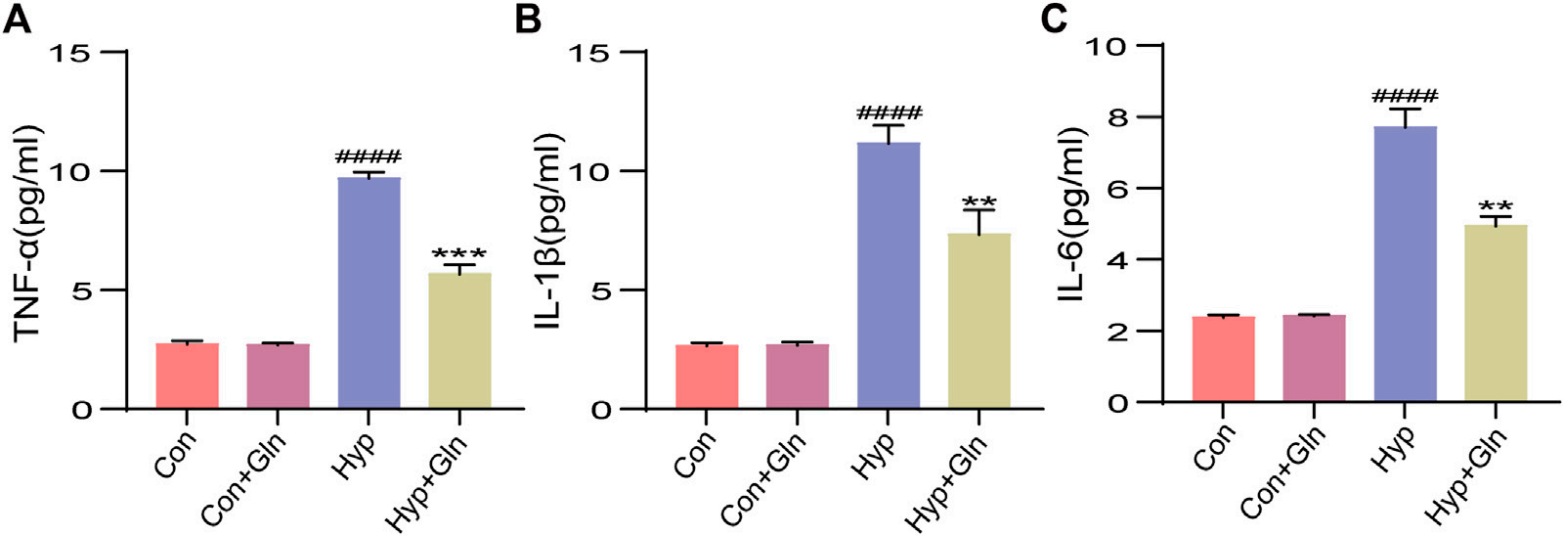
Gln inhibits inflammation caused by hyperoxia. **(A)** TNF-α content in hippocampus. Con (2.73 ± 0.84), Con + Gln (2.69 ± 0.41), Hyp (9.70 ± 0.15), Hyp + Gln (5.67 ± 0.22). **(B)** IL-1β content in hippocampus. Con (2.65 ± 0.08), Con + Gln (2.68 ± 0.08), Hyp (11.16 ± 0.44), Hyp + Gln (7.33 ± 0.60). **(C)** IL-6 content in hippocampus. Con (2.38 ± 0.05), Con + Gln (2.42 ± 0.02), Hyp (7.71 ± 0.30), Hyp + Gln (4.94 ± 0.16) (*n* = 3). ^####^ *p* < 0.0001 vs. the Con, **p < 0.01, ***p < 0.001 vs. the Hyp. *p* < 0.05 was considered statistically significant.

To verify whether Gln could inhibit the inflammatory response caused by hyp, we examined the expression of TNF-α (Figure 5A), IL-1β (Figure 5B), and IL-6 (Figure 5C) in hippocampal tissues. Exposure to hyp significantly increased the expression levels of TNF-α (*p* < 0.0001), IL-1β (*p* < 0.0001), and IL-6 (*p* < 0.0001), showing that hyp induces an inflammatory response. However, these changes were significantly reversed after Gln application (TNF-α: *p* < 0.001, IL-1β: *p* < 0.01, IL-6: *p* < 0.01). TNF-α: Con (2.73 ± 0.84), Con + Gln (2.69 ± 0.41), Hyp (9.70 ± 0.15), Hyp + Gln (5.67 ± 0.22). IL-1β: Con (2.65 ± 0.08), Con + Gln (2.68 ± 0.08), Hyp (11.16 ± 0.44), Hyp + Gln (7.33 ± 0.60). IL-6: Con (2.38 ± 0.05), Con + Gln (2.42 ± 0.02), Hyp (7.71 ± 0.30), Hyp + Gln (4.94 ± 0.16) (*n* = 3).

### Gln inhibits neuronal apoptosis in rats with hyperoxia-induced brain injury

**FIGURE 6:**
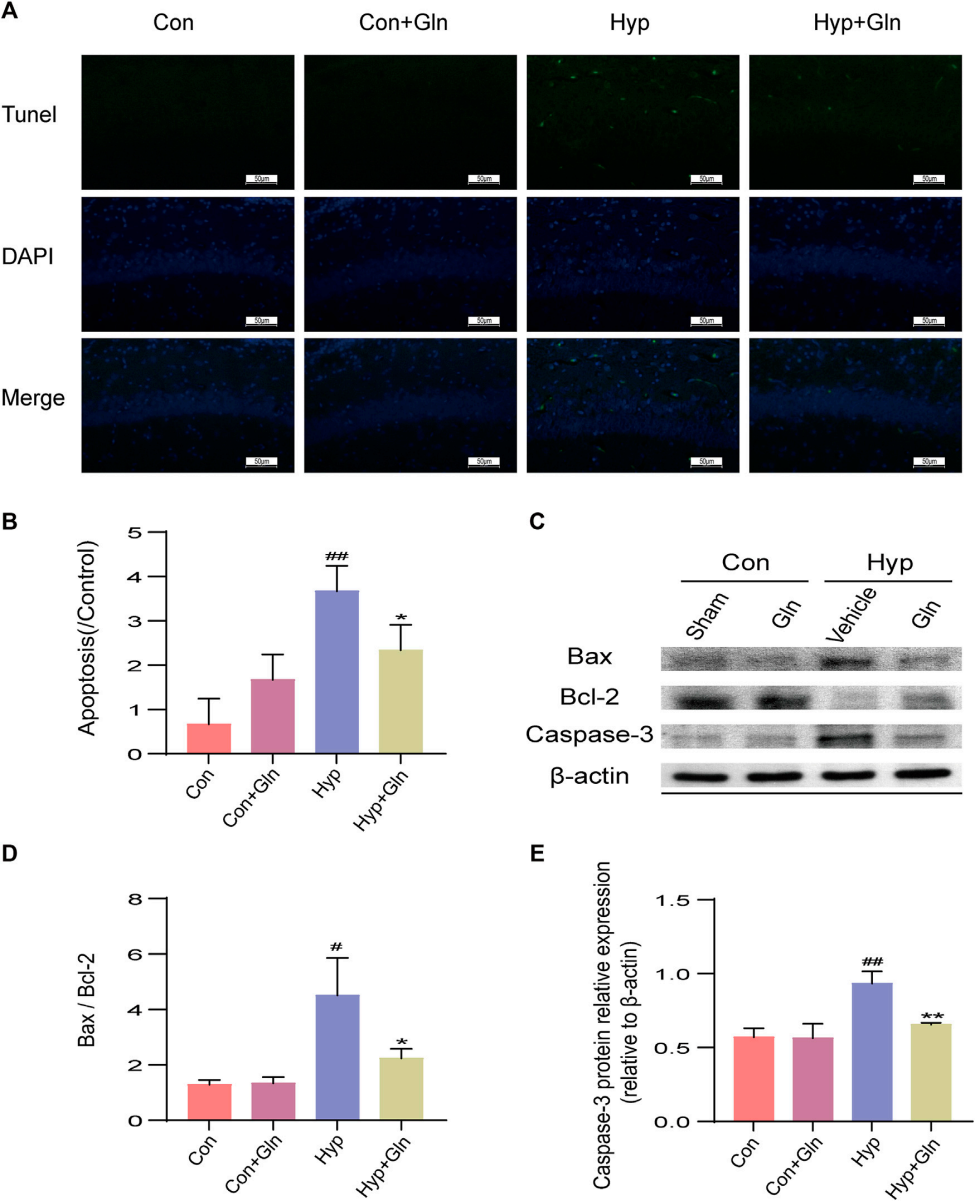
Gln inhibits neuronal apoptosis in rats with hyperoxia-induced brain injury. **(A)** TUNEL staining of hippocampal tissue. Scale bar = 50 µm **(B)** Hippocampal Apoptosis Index (AI). Con (0.67 ± 0.33), Con + Gln (1.67 ± 0.33), Hyp (3.67 ± 0.33), Hyp + Gln (2.33 ± 0.33) **(C)** Representative images of Western blotting analysis of Bax, Bcl-2 and Caspase-3 of each group. **(D)** Ratio of Bcl-2 to Bax. Con (1.29 ± 0.10), Con + Gln (1.34 ± 0.13), Hyp (4.50 ± 0.79), Hyp + Gln (2.24 ± 0.20). **(E)** Caspase-3 content in hippocampus. Con (0.57 ± 0.04), Con + Gln (0.56 ± 0.06), Hyp (0.93 ± 0.05), Hyp + Gln (0.66 ± 0.01) (*n* = 3). ^#^ *p* < 0.05, ^##^ *p* < 0.01 vs. the Con, *p < 0.05, **p < 0.01 vs. the Hyp. *p* < 0.05 was considered statistically significant.

TUNEL staining was performed to evaluate the inhibitory effect of Gln on apoptosis (Figure 6A). The results showed that the number of TUNEL-positive cells increased significantly after exposure to hyp (*p* < 0.01), while Gln administration exerted an inhibitory effect on hyp-induced apoptosis (*p* < 0.05) (Figure 6B). Con (0.67 ± 0.33), Con + Gln (1.67 ± 0.33), Hyp (3.67 ± 0.33), Hyp + Gln (2.33 ± 0.33) (*n* = 3).

To further clarify the apoptosis of hippocampal tissues in each group, caspase-3, Bax and Bcl-2 was determined by western blotting (Figure 6C). The results showed that the Bax/Bcl-2 ratio (*p* < 0.05) and caspase-3 content (*p* < 0.01) were significantly increased in the Hyp group; in contrast, it considerably improved in the Hyp + Gln group (Bax/Bcl-2: *p* < 0.05; Caspase-3: *p* < 0.01) (Figures 6D, E). Bax/Bcl-2: Con (1.29 ± 0.10), Con + Gln (1.34 ± 0.13), Hyp (4.50 ± 0.79), Hyp + Gln (2.24 ± 0.20); Caspase-3: Con (0.57 ± 0.04), Con + Gln (0.56 ± 0.06), Hyp (0.93 ± 0.05), Hyp + Gln (0.66 ± 0.01) (*n* = 3).

### Gln increases BDNF, Synapsin-1 and MBP expression in hippocampal tissue of rats with hyp-induced brain injury

**FIGURE 7:**
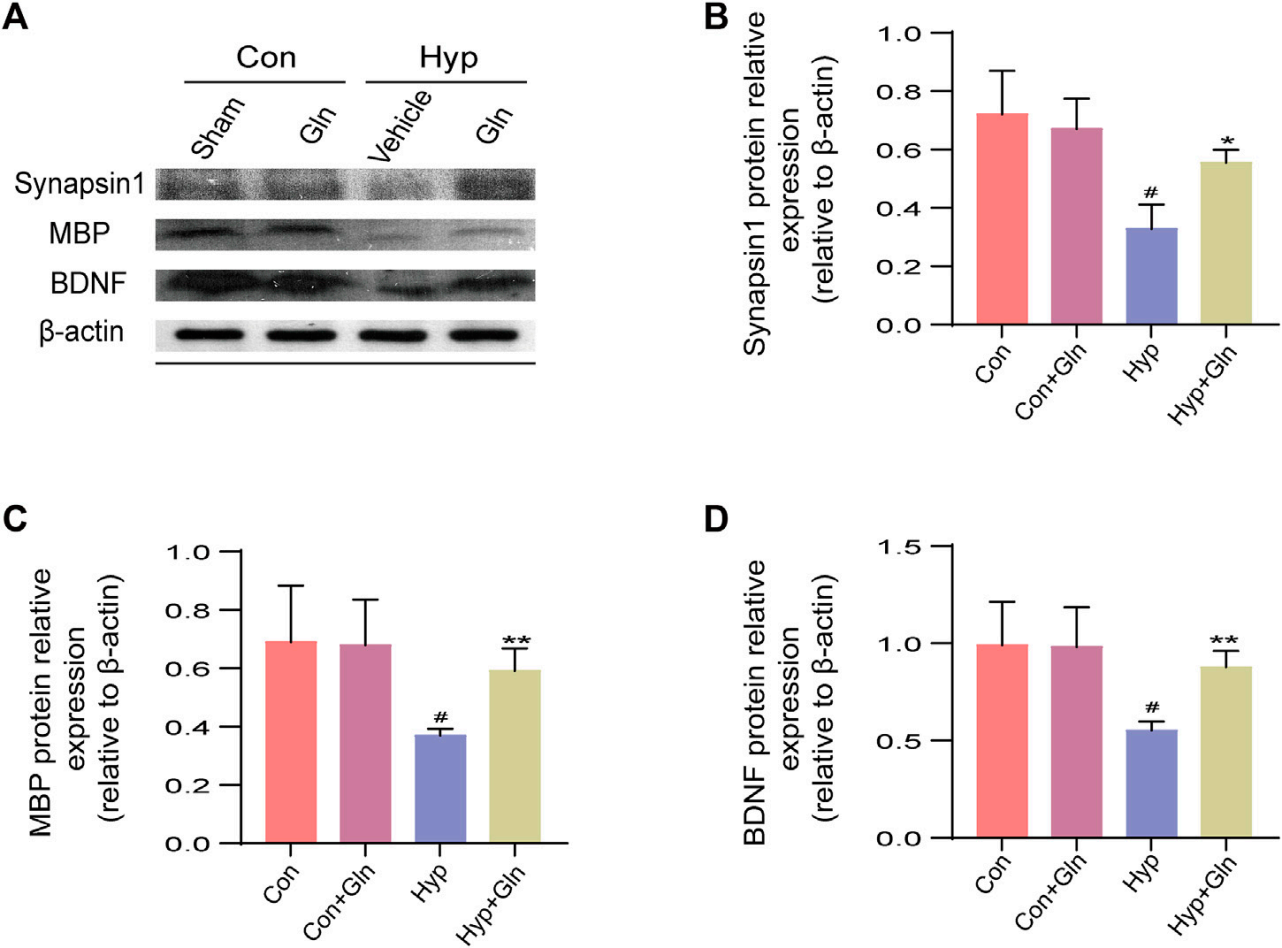
Gln increases BDNF, synapsin-1 and MBP expression in hippocampal tissue of rats with hyperoxia-induced brain injury. **(A)** Representative images of Western blotting analysis of synapsin-1, MBP and BDNF of each group. **(B)** Synapsin-1 content in hippocampus. Con (0.72 ± 0.09), Con + Gln (0.67 ± 0.06), Hyp (0.33 ± 0.05), Hyp + Gln (0.55 ± 0.03). **(C)** MBP content in hippocampus. Con (0.69 ± 0.11), Con + Gln (0.68 ± 0.09), Hyp (0.37 ± 0.01), Hyp + Gln (0.59 ± 0.04). **(D)** BDNF content in hippocampus. Con (0.99 ± 0.13), Con + Gln (0.98 ± 0.12), Hyp (00.55 ± 0.03), Hyp + Gln (0.88 ± 0.05). (*n* = 3). ^#^ *p* < 0.05 vs. the Con, *p < 0.05, **p < 0.01 vs. the Hyp. *p* < 0.05 was considered statistically significant.

To evaluate whether the neuroprotective effect of Gln is related to synaptic plasticity, impaired myelin formation, and the absence of neurotrophic factors, the expression of brain-derived neurotrophic factor (BDNF), synapsin I, and myelin basic protein (MBP) was examined after homogenizing hippocampal tissue obtained from each group of rats (Figure 7A). The results showed that the expression of synapsin I (*p* < 0.05), MBP (*p* < 0.05) and BDNF (*p* < 0.05) proteins decreased significantin the Hyp group. In the Hyp + Gln group, the expression of these proteins was significantly higher than that in the Hyp group (synapsin I: *p* < 0.05; MBP: *p* < 0.01; BDNF: *p* < 0.01). This indicated that Gln increased synapsin I (Figure 7B), MBP (Figure 7C) and BDNF (Figure 7D) expression in the hippocampal tissue of rats with hyp-induced brain injury to improve neural outcomes. Synapsin-1: Con (0.72 ± 0.09), Con + Gln (0.67 ± 0.06), Hyp (0.33 ± 0.05), Hyp + Gln (0.55 ± 0.03); MBP: Con (0.69 ± 0.11), Con + Gln (0.68 ± 0.09), Hyp (0.37 ± 0.01), Hyp + Gln (0.59 ± 0.04); BDNF: Con (0.99 ± 0.13), Con + Gln (0.98 ± 0.12), Hyp (00.55 ± 0.03), Hyp + Gln (0.88 ± 0.05) (*n* = 3).

### Gln amelioration of hyp-induced brain injury may be related to the MKP-1/MAPK signaling pathway

**FIGURE 8:**
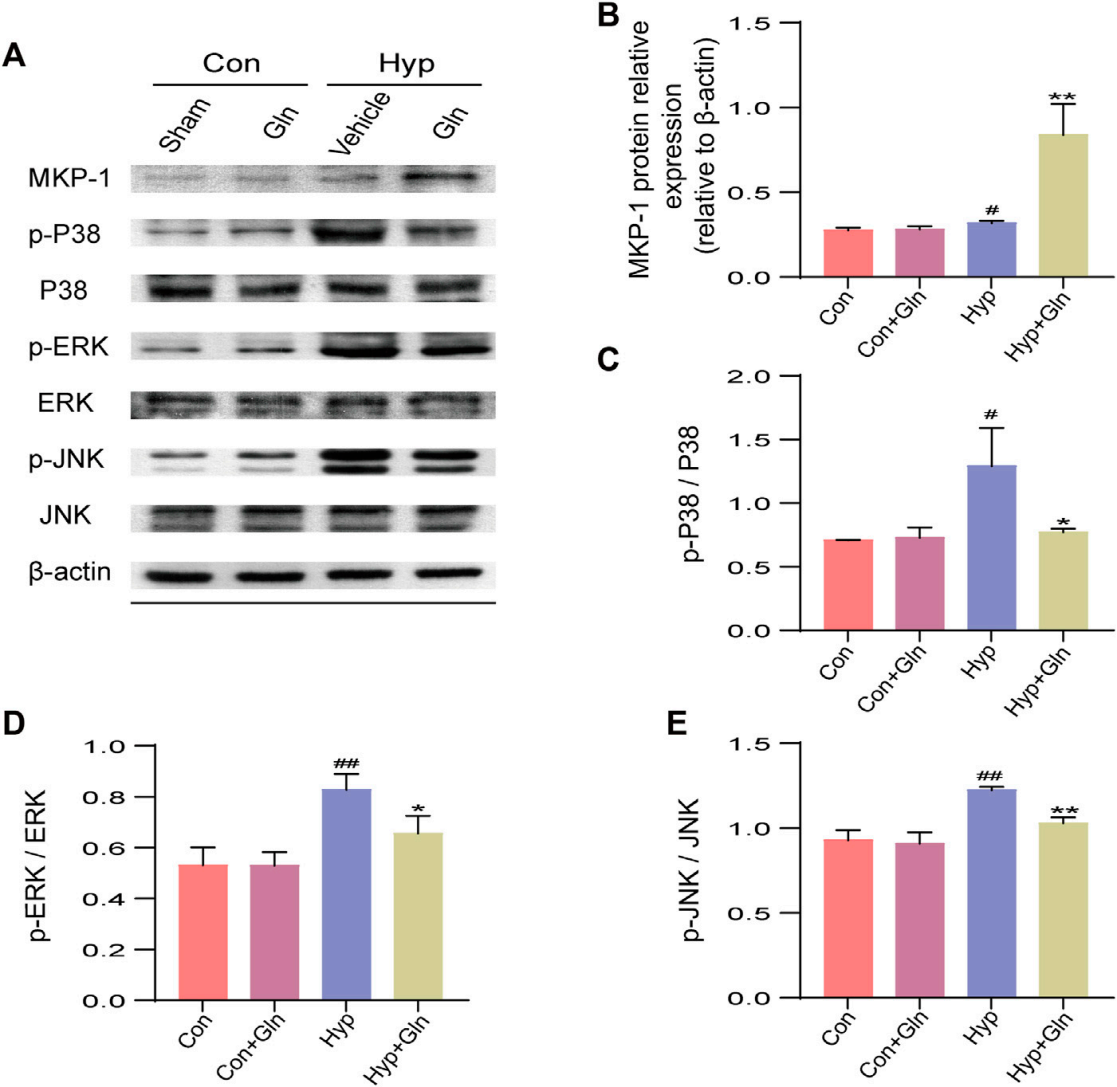
Gln amelioration of hyperoxia-induced brain injury may be related to MKP-1/MAPK signaling pathway. **(A)** Representative images of Western blotting analysis of MKP-1, p-p38, p38, p-ERK, ERK, p-JNK, and JNK of each group. **(B)** Quantification of MKP-1. Con (0.28 ± 0.01), Con + Gln (0.28 ± 0.01), Hyp (0.32 ± 0.01), Hyp + Gln (0.84 ± 0.11). **(C)** Ratio of p-p38 to p38. Con (0.71 ± 0.00), Con + Gln (0.73 ± 0.05), Hyp (1.29 ± 0.17), Hyp + Gln (0.77 ± 0.02). **(D)** Ratio of p-ERK to ERK. Con (0.53 ± 0.04), Con + Gln (0.53 ± 0.03), Hyp (0.83 ± 0.04), Hyp + Gln (0.66 ± 0.04). **(E)** Ratio of p-JNK to JNK. Con (0.93 ± 0.03), Con + Gln (0.91 ± 0.04), Hyp (1.23 ± 0.01), Hyp + Gln (1.03 ± 0.02). (*n* = 3). ^#^ *p* < 0.05, ^##^ *p* < 0.01 vs. the Con, *p < 0.05, **p < 0.01 vs. the Hyp. *p* < 0.05 was considered statistically significant.

The MAPK signaling pathway is closely associated with oxidative stress, inflammation, and apoptosis. To assess the role of Gln in a model of hyp-induced brain injury and its association with the MKP-1/MAPK signaling pathway, we examined the expression of MKP-1/MAPK signaling pathway-related proteins (Figure 8A). The results showed that compared with the Con group, MKP-1 expression (*p* < 0.05) (Figure 8B) was slightly increased, and p-p38/p38 (*p* < 0.05) (Figure 8C), p-ERK/ERK (*p* < 0.01) (Figure 8D), and p-JNK/JNK (*p* < 0.01) (Figure 8E) expression was significantly decreased in the Hyp group. Compared with the Hyp group, the Hyp + Gln group showed increased MKP-1 protein expression (*p* < 0.01) and significantly decreased p-p38/p38 (*p* < 0.05), p-ERK/ERK (*p* < 0.05), and p-JNK/JNK (*p* < 0.01) expression. MKP-1: Con (0.28 ± 0.01), Con + Gln (0.28 ± 0.01), Hyp (0.32 ± 0.01), Hyp + Gln (0.84 ± 0.11); p-p38/p38: Con (0.71 ± 0.00), Con + Gln (0.73 ± 0.05), Hyp (1.29 ± 0.17), Hyp + Gln (0.77 ± 0.02); p-ERK/ERK: Con (0.53 ± 0.04), Con + Gln (0.53 ± 0.03), Hyp (0.83 ± 0.04), Hyp + Gln (0.66 ± 0.04); p-JNK/JNK: Con (0.93 ± 0.03), Con + Gln (0.91 ± 0.04), Hyp (1.23 ± 0.01), Hyp + Gln (1.03 ± 0.02) (n = 3).

### Gln ameliorates neurobehavioral deficits in rats with hyp-induced brain injury

**FIGURE 9:**
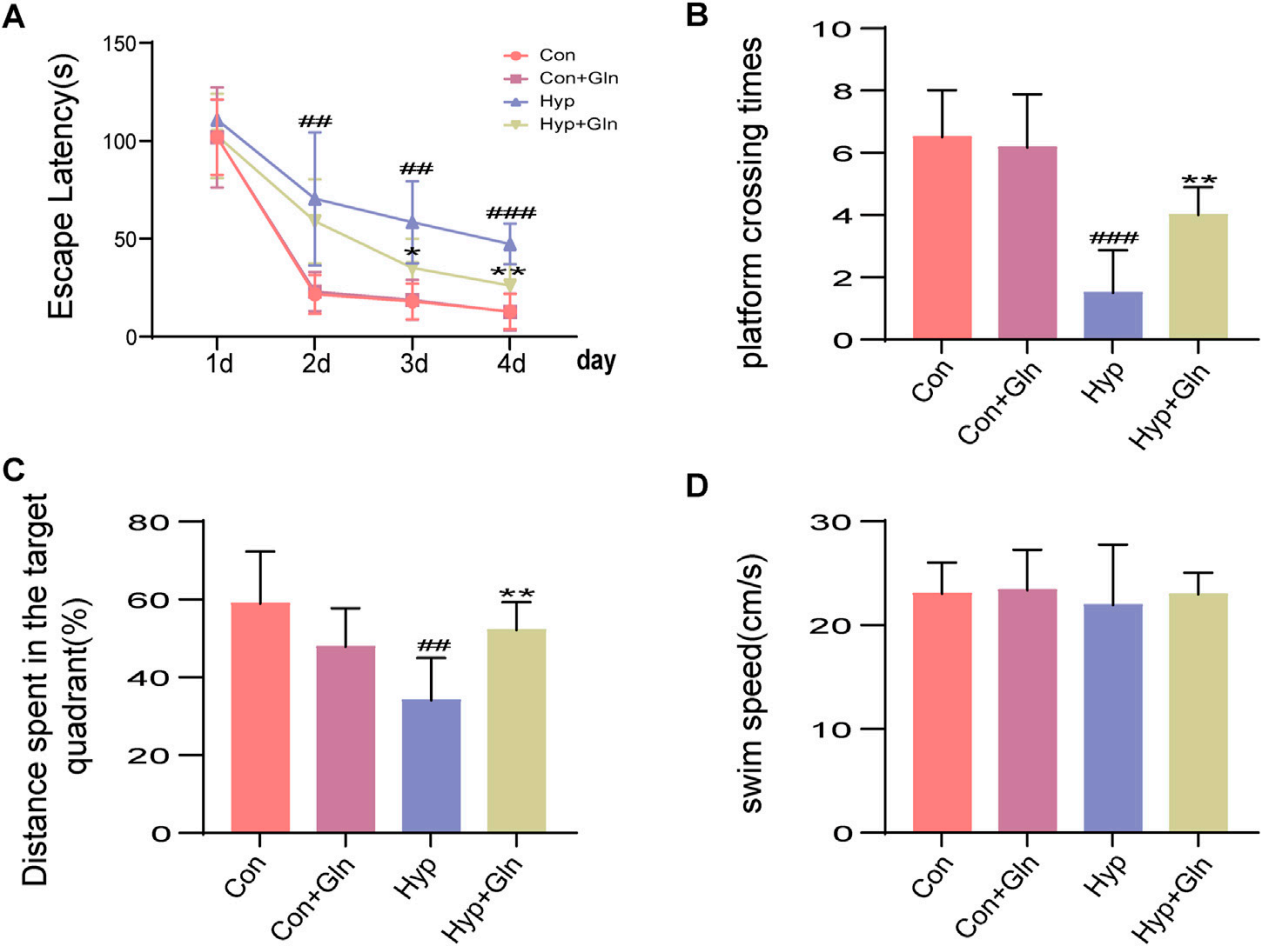
Gln ameliorates distant neurobehavioral deficits in rats with hyperoxia-induced brain injury. **(A)** Latency to escape in MWM. Con (101.96 ± 7.86, 21.63 ± 4.05, 18.07 ± 3.71, 12.94 ± 3.68), Con + Gln (101.76 ± 10.43, 23.10 ± 4.09, 18.85 ± 4.16, 12.66 ± 3.85), Hyp (110.78 ± 4.19, 70.39 ± 13.85, 58.48 ± 8.53, 47.28 ± 4.24), Hyp + Gln (102.54 ± 8.82, 58.89 ± 8.76, 35.25 ± 5.99, 26.22 ± 4.50). **(B)** Times crossing platform in MWM. Con (6.50 ± 0.62), Con + Gln (6.17 ± 0.70), Hyp (1.50 ± 0.56), Hyp + Gln (4.00 ± 0.37). **(C)** Distance spend in the target quarter in MWM. Con (58.97 ± 5.46), Con + Gln (47.82 ± 4.06), Hyp (34.12 ± 4.42), Hyp + Gln (52.19 ± 2.92). **(D)** Swim speed in MWM. Con (23.04 ± 1.22), Con + Gln (23.39 ± 1.58), Hyp (21.95 ± 2.37), Hyp + Gln (22.96 ± 0.86) (*n* = 6). ^##^ *p* < 0.01, ^###^ *p* < 0.001 vs. the Con, *p < 0.05, **p < 0.01 vs. the Hyp. *p* < 0.05 was considered statistically significant.

The Morris water maze assesses spatial learning and long-term memory. To further assess the neuroprotective effect of Gln, the neurobehavioral function of each group of adolescent rats was examined using the MWM experiment (P30-34). The escape latency (Figure 9A) showed a decreasing trend with increasing time in all groups. Gln administration failed to shorten escape latency after hyp-induced injury on the first day of testing. However, the mean escape latency was significantly shorter (*p* < 0.05; *p* < 0.01) in the Hyp + Gln group from Days three to four. These results suggest that rats in all groups had the ability to learn and remember. These abilities were impaired to varying degrees by hyp-induced brain injury; however, Gln improved the spatial learning abilities of rats with hyp-induced brain injury. In the observation of the swimming path on Day 5, it was found that the Hyp group rats crossed the platform position (Figure 9B) less (*p* < 0.001) often than the Con group did, and the percentage of distance traveled in the target quadrant (Figure 9C) was less (*p* < 0.01) than that of the Con group. In the Hyp + Gln group, the number of platform crossings increased (*p* < 0.01) compared to that in the Hyp group, and the percentage of distance traveled in the target quadrant increased (*p* < 0.01) compared to that in the Hyp group. These results suggest that hyp-induced brain damage during the neonatal period affects memory capacity in adolescent rats. However, administration of Gln significantly improved the duration spent in the plateau quadrant, suggesting that Gln effectively alleviated memory impairment induced by hyp. No significant difference was observed in the mean swimming speed between groups (Figure 9D). Latency to escape: Con (101.96 ± 7.86, 21.63 ± 4.05, 18.07 ± 3.71, 12.94 ± 3.68), Con + Gln (101.76 ± 10.43, 23.10 ± 4.09, 18.85 ± 4.16, 12.66 ± 3.85), Hyp (110.78 ± 4.19, 70.39 ± 13.85, 58.48 ± 8.53, 47.28 ± 4.24), Hyp + Gln (102.54 ± 8.82, 58.89 ± 8.76, 35.25 ± 5.99, 26.22 ± 4.50); Times crossing platform: Con (6.50 ± 0.62), Con + Gln (6.17 ± 0.70), Hyp (1.50 ± 0.56), Hyp + Gln (4.00 ± 0.37); Distance spend in the target quarter: Con (58.97 ± 5.46), Con + Gln (47.82 ± 4.06), Hyp (34.12 ± 4.42), Hyp + Gln (52.19 ± 2.92); Swim speed: Con (23.04 ± 1.22), Con + Gln (23.39 ± 1.58), Hyp (21.95 ± 2.37), Hyp + Gln (22.96 ± 0.86) (n = 6).

## Discussion

Long-term hyperoxia treatment can lead to behavioral abnormalities and neurocognitive and learning deficits in preterm infants, which is thought to be related to damage to hippocampal neurons caused by oxidative stress, inflammation, apoptosis, and reduced expression of neurotrophic factors (Sifringer et al., 2015). Spatial cognition and memory are inseparable from hippocampal function, and alterations in hippocampal structure and function are associated with reduced spatial, social, and mathematical abilities (Banker et al., 2021). We found that hyperoxia caused brain tissue edema and hippocampal damage in neonatal rats, with significant deficits in spatial memory and learning ability observed during adolescence. Gln is an *in vivo* antioxidant precursor substance with multiple regulatory mechanisms and known antioxidant potential (Cetinbas et al., 2010), and its neuroprotective potential has been demonstrated in animal models of neonatal brain injury, including hypoxia-induced neuroinflammation. Gln ameliorates spatial learning and memory dysfunction in neonatal brain injury caused by hyperoxia. The neuroprotective effects of Gln appear to be associated with its anti-inflammatory and anti-apoptotic effects after attenuation of oxidative stress, which is beneficial for long-term brain development (de Kieviet et al., 2012). Thus, Gln is a promising drug for the treatment of hyp-induced brain injury.

Oxidative stress is a key factor in the complex cascade of brain injury mechanisms. In preterm infants, whose antioxidant systems are not yet well-developed, the excessive ROS generated in a hyperoxic environment trigger an inflammatory response that induces prostaglandin production and leads to the formation of intercellular interstitial edema (Lapi et al., 2020). Microglia, a major component of the immune defense system of the central nervous system, are overactivated by multiple diseases and external stimuli (including traumatic brain trauma and neurological infections) (Loane and Byrnes, 2010), and in response secrete ROS to induce oxidative stress and inflammatory responses, which can lead to further neurological diseases. It was found that increased levels of pro-inflammatory cytokines in the hippocampus, such as IL-6, IL-1β, and TNF-α, activate microglia and lead to neurotransmitter alterations, causing dysfunction in spatial learning and cognitive faculties (Hernández-Rabaza et al., 2016). Gln, a precursor of the endogenous antioxidant glutathione, has antioxidant potential and the ability to balance oxidative stress (Cetinbas et al., 2010). It may exert neuroprotective effects through inhibition of excessive inflammatory responses. In this study, administration of Gln was seen to significantly reduce hyp-induced edema in brain tissue, ameliorate hippocampal damage, decrease microglial activation, significantly decrease MDA and ROS levels, and significantly increase SOD and GSH levels. The results suggest that in the developing brain, Gln may have antioxidant activity against hyp-induced oxidative stress, inhibit microglial overactivation, and inhibit the synthesis and release of pro-inflammatory cytokines, thus exerting a protective effect by reducing neuronal inflammation and damage.

Many proteins also play important roles in the development and treatment of brain injury. BDNF is an important protein that can influence neuroplasticity, affecting cognition, brain structure and function (Roy et al., 2020). Synapsin I, the most abundant protein in synaptic vesicles, is involved in synaptic development and transmission, regulation of neurotransmitter release (Lu et al., 2018), neurodevelopment, and neuronal growth. It plays a key role in information processing and transmission and is thought to have a positive impact on learning and memory consolidation (Zhu et al., 2018). MBP is the main protein in the myelin sheath of the central nervous system, where it maintains sheath stability, structure, and function (Krugmann et al., 2020). MBP is involved in the myelination process as a major component of the myelin membrane in the CNS, and changes in MBP levels may reflect the degree of white matter damage in astrocytes, which has been identified as a sensitive and specific marker of brain damage in recent years (Zhou et al., 2015). Oxidative stress, inflammatory responses, and apoptosis triggered by hyperoxia in the developing brain are believed to have a major effect on the survival of immature oligodendrocytes (which are highly sensitive to oxygen concentration), leading to impaired myelin formation (Brehmer et al., 2012). We found that hyperoxia leads to decreased levels of BDNF, synapsin I, and MBP in the hippocampus, in agreement with previous findings (Kim et al., 2016). However, Gln may also play a therapeutic role by increasing neurotrophic factor levels and regulating signals related to synaptic plasticity.

MAPK mediates cellular responses to external stimuli and regulates a variety of cellular activities such as cell proliferation, differentiation, apoptosis, and neuronal plasticity (Keshet and Seger, 2010). Evidence suggests that the MAPK signaling pathway can regulate oxidative stress (Chen et al., 2020) and microglial activation (Na et al., 2014) to suppress inflammatory factor production. It is also involved in apoptosis (Hsieh et al., 2021). Negative regulation of MAPK activity is achieved through MKP. MKP-1 expression is upregulated both when oxidative damage occurs (Patterson et al., 2009) and on administration of neuroprotective drugs (Jeanneteau et al., 2010). MKP-1 is known to reduce levels of pro-inflammatory cytokines (Zhang et al., 2012) and apoptosis by accelerating MAPK inactivation, thus improving neuronal function by decreasing phosphorylation of JNK, ERK, and p38 (Horita et al., 2010). Gu et al. showed that Gln inhibited ROS production, increased antioxidant enzyme activity, and ameliorated intestinal inflammation and oxidative damage by reducing the activation of the MAPK pathway. In keeping with previous findings, we found that the administration of Gln increased MKP-1 expression, inhibited phosphorylation of JNK, ERK, and p38, and reduced oxidative stress, inflammation, and apoptosis. Therefore, we speculate that Gln may induce MKP-1 and thereby inhibit activation of the MAPK signaling pathway, leading to a reduction in oxidative stress, inflammation, and apoptosis in cells and attenuating hyp-induced damage to cerebral tissue.

## Conclusion

**FIGURE 10:**
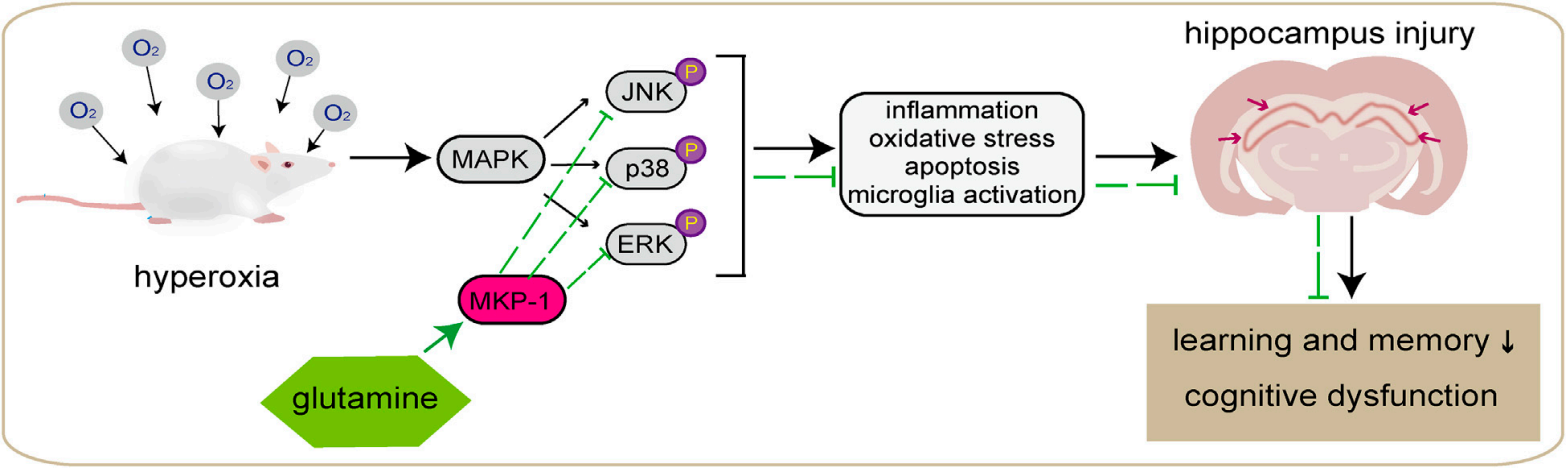
Graphical abstract. Hyperoxia can lead to behavioral abnormalities and neurocognitive and learning deficits in newborn rats, which is thought to be related to damage to hippocampal neurons caused by oxidative stress, inflammation, apoptosis, and microglial overactivation. Gln may induce MKP-1 and thereby inhibit activation of the MAPK signaling pathway, leading to a reduction in oxidative stress, inflammation, and apoptosis in cells and attenuating hyperoxia-induced damage to cerebral tissue, and improve learning and memory dysfunction.

This study shows that in newborn rats with hyp-induced brain injury, Gln has potent anti-inflammatory and antioxidant activities, reduces hippocampal damage and neurological dysfunction, ameliorates hyp-induced brain injury, and decreases apoptosis, thus alleviating learning difficulties and memory dysfunction in newborn rats with hyp-induced brain injury. Our results suggest that the molecular mechanism underlying these effects may be related to the MKP-1/MAPK signaling pathway (Figure 10). This study provides a theoretical basis for the possible pathological mechanisms underlying hyp-induced newborn neurological injury and its prevention. In summary, Gln has a neuroprotective effect in neonatal brain injury caused by hyperoxia and has potential as a drug candidate.

## Data Availability

The original contributions presented in the study are included in the article/supplementary material, further inquiries can be directed to the corresponding authors.
